# Supplementary material for: Crowdsourcing digital health measures to predict Parkinson’s disease severity: the Parkinson’s Disease Digital Biomarker DREAM Challenge
Source: NPJ Digit Med. 2021 Mar 19;4:53. doi: 10.1038/s41746-021-00414-7 (PMC7979931; doi:10.1038/s41746-021-00414-7)
Supplement: Supplementary file 2 — Reporting Summary [file 41746_2021_414_MOESM2_ESM.pdf]

nature research

Corresponding author(s): Sieberts

Last updated by author(s): Jan 14, 2021

Reporting Summary

Nature Research wishes to improve the reproducibility of the work that we publish. This form provides structure for consistency and transparency in reporting. For further information on Nature Research policies, see our Editorial Policies and the Editorial Policy Checklist.

Statistics

For all statistical analyses, confirm that the following items are present in the figure legend, table legend, main text, or Methods section.

n/a	Confirmed	
☐	☒	The exact sample size (*n*) for each experimental group/condition, given as a discrete number and unit of measurement
☐	☒	A statement on whether measurements were taken from distinct samples or whether the same sample was measured repeatedly
☐	☒	The statistical test(s) used AND whether they are one- or two-sided<br>*Only common tests should be described solely by name; describe more complex techniques in the Methods section.*
☐	☒	A description of all covariates tested
☐	☒	A description of any assumptions or corrections, such as tests of normality and adjustment for multiple comparisons
☒	☐	A full description of the statistical parameters including central tendency (e.g. means) or other basic estimates (e.g. regression coefficient) AND variation (e.g. standard deviation) or associated estimates of uncertainty (e.g. confidence intervals)
☒	☐	For null hypothesis testing, the test statistic (e.g. *F*, *t*, *r*) with confidence intervals, effect sizes, degrees of freedom and *P* value noted<br>*Give P values as exact values whenever suitable.*
☒	☐	For Bayesian analysis, information on the choice of priors and Markov chain Monte Carlo settings
☒	☐	For hierarchical and complex designs, identification of the appropriate level for tests and full reporting of outcomes
☐	☒	Estimates of effect sizes (e.g. Cohen's *d*, Pearson's *r*), indicating how they were calculated

Our web collection on statistics for biologists contains articles on many of the points above.

Software and code

Policy information about availability of computer code

Data collection	Data for mPower was collected via an iPhone app: https://apps.apple.com/us/app/parkinson-mpower-2/id1375781575<br>The L-dopa data were collected via the GENEActiv and Pebble Operating Systems.
Data analysis	Code used to evaluate submissions are available through https://github.com/Sage-Bionetworks/PDbiomarkerChallengeScoring. Methods descriptions and code for all available submissions is available through Synapse (doi: 10.7303/syn8717496).

For manuscripts utilizing custom algorithms or software that are central to the research but not yet described in published literature, software must be made available to editors and reviewers. We strongly encourage code deposition in a community repository (e.g. GitHub). See the Nature Research guidelines for submitting code & software for further information.

Data

Policy information about availability of data

All manuscripts must include a data availability statement. This statement should provide the following information, where applicable:

- Accession codes, unique identifiers, or web links for publicly available datasets
- A list of figures that have associated raw data
- A description of any restrictions on data availability

Data, predictions, feature scores, and methods descriptions used and generated in this challenge are available through Synapse (doi: 10.7303/syn8717496). The mPower (doi: 10.7303/syn4993293) and MJFF Levodopa Response Study (doi: 10.7303/syn20681023) data are also available.

April 2020

Field-specific reporting

Please select the one below that is the best fit for your research. If you are not sure, read the appropriate sections before making your selection.

☒ Life sciences ☐ Behavioural & social sciences ☐ Ecological, evolutionary & environmental sciences

For a reference copy of the document with all sections, see nature.com/documents/nr-reporting-summary-flat.pdf

Life sciences study design

All studies must disclose on these points even when the disclosure is negative.

Sample size	All available data from these previously collected studies were leveraged.
Data exclusions	NA
Replication	Models were assessed on a held-out portion of the data from the same data set. The labels of the test data were only available to challenge organizers, not modelers to enable unbiased assessment.
Randomization	NA
Blinding	NA

Reporting for specific materials, systems and methods

We require information from authors about some types of materials, experimental systems and methods used in many studies. Here, indicate whether each material, system or method listed is relevant to your study. If you are not sure if a list item applies to your research, read the appropriate section before selecting a response.

Materials & experimental systems

n/a	Involved in the study	
☒	☐	Antibodies
☒	☐	Eukaryotic cell lines
☒	☐	Palaeontology and archaeology
☒	☐	Animals and other organisms
☐	☒	Human research participants
☒	☐	Clinical data
☒	☐	Dual use research of concern

Methods

n/a	Involved in the study	
☒	☐	ChIP-seq
☒	☐	Flow cytometry
☒	☐	MRI-based neuroimaging

Human research participants

Policy information about studies involving human research participants

Population characteristics	mPower enrolled subjects at least 18 who owned an iPhone.<br>L-Dopa enrolled subjects with Parkinson's Disease who exhibit motor fluctuations.
Recruitment	The mPower Study was conducted remotely through an iPhone application. Participants provided consent through an interactive e-consent process, which included a quiz evaluating their understanding of the consent provided. The study and consent procedure were approved by the Western Institutional Review Board (WIRB 20181960).<br>Levodopa Response Study subjects were recruited and enrolled at two study sites: Spaulding Rehabilitation Hospital and Mount Sinai Hospital. All subjects signed an informed consent form. All procedures were approved by the Institutional Review Board of both study sites (Spaulding Rehabilitation Hospital IRB # 2014P000847; Mount Sinai Hospital IRB # 14-1569).
Ethics oversight	The mPower Study was conducted remotely through an iPhone application. Participants provided consent through an interactive e-consent process, which included a quiz evaluating their understanding of the consent provided. The study and consent procedure were approved by the Western Institutional Review Board (WIRB 20181960).<br>Levodopa Response Study subjects were recruited and enrolled at two study sites: Spaulding Rehabilitation Hospital and Mount Sinai Hospital. All subjects signed an informed consent form. All procedures were approved by the Institutional Review Board of both study sites (Spaulding Rehabilitation Hospital IRB # 2014P000847; Mount Sinai Hospital IRB # 14-1569).

Note that full information on the approval of the study protocol must also be provided in the manuscript.
